# Psychometric Properties of the Japanese Translation of the Parent Overprotection Measure for Mother and Father Reports

**DOI:** 10.1007/s10578-024-01753-8

**Published:** 2024-09-09

**Authors:** Sho Okawa, Ronald M. Rapee, Takahito Takahashi, Tessa Reardon, Honami Arai, Eiji Shimizu, Cathy Creswell

**Affiliations:** 1 Department of Experimental Psychology, University of Oxford, Anna Watts Building, Radcliffe Observatory Quarter, Woodstock Road, Oxford, OX2 6GG UK; 2 Research Center for Child Mental Development, Chiba University, 1-8-1, Inohana, Chuo Ku, Chiba-Shi, Chiba, 260-8670 Japan; 3 Department of Life Sciences, The University of Tokyo, 3-8-1, Komaba, Meguro-Ku, Tokyo, 153-8902 Japan; 4 Centre for Lifespan Health and Wellbeing, School of Psychological Sciences, Macquarie University, Balaclava Road, 2109, North Ryde, NSW Australia; 5 Faculty of Education, University of Miyazaki, 1-1 Gakuenkibanadai-Nishi, Miyazaki , 889-2192 Japan; 6 Department of Psychiatry, University of Oxford, Warneford Hospital, Oxford, OX3 7JX UK; 7 Center for Research on Counseling and Support Services, Tokyo University, 7-3-1, Hongo, Bunkyo-Ku, Tokyo, 113-0033 Japan; 8 Department of Cognitive Behavioral Physiology, Graduate School of Medicine, Chiba University, 1-8-1, Inohana, Chuo Ku, Chiba-Shi, Chiba, 260-8670 Japan

**Keywords:** Childhood anxiety, Parent, Overprotection, Japan

## Abstract

The Parent Overprotection Measure (POM) is a promising scale to measure parent overprotection toward a child from the parent’s perspective. However, no Japanese translation of the scale has been developed, and whether the POM can be applied to a Japanese population is unknown. This study translated the POM into Japanese and examined its psychometric properties. Parents of 380 children aged 4 to 7 years (including 190 mothers and 190 fathers) completed online questionnaires. Exploratory and confirmatory factor analyses (CFA) indicated that the Japanese translation of the POM has a bi-factor structure, including one general factor (general overprotection) and two specific factors (care/attention and control/prevention). The measurement invariance of reports from mothers’ and fathers’ perspectives was confirmed by multiple group CFA. The McDonald’s Omega was acceptable for all factors, but the general overprotection factor explained most scale variance. Pearson’s correlation coefficients were more than .20 between the control/prevention factor and child anxiety symptoms in both mother and father reports. The correlation between the control/prevention factor and parent anxiety according to fathers’ reports also exceeded .20. These results provided the factor structure and supported the reliability of the POM among a Japanese population; however, further investigation of the validity of the scale is needed.

**Supplementary Information:**

The online version contains supplementary material available at 10.1007/s10578-024-01753-8.

## Introduction

Child anxiety can be a problem if it is excessive and interferes with the child’s daily life [1]. Lifetime anxiety disorders often start in childhood with the first peak of the onset of anxiety disorders at 5.5 years [2]. Anxiety disorders in childhood may become chronic and can impact a wide range of factors relevant to quality of life, including eating disorders, physical health, poor education, poor employment, etc. [3]. Therefore, early assessment of potential risk factors for the development of anxiety disorders is essential to prevent lifetime anxiety problems.

A number of factors have been demonstrated to increase risk for the development and maintenance of child anxiety disorders, including genetic and temperamental vulnerability, social and environmental factors, and internal, psychological factors (e.g., [4–6]). Among the social and environmental factors of relevance to childhood anxiety disorders, a range of parental behaviours have been implicated [6–8]. Foremost among these is parent overprotection, which has been found to be associated with anxiety disorders in children and adolescents [9, 10]. Parent overprotection is defined as parents’ excessively cautious behaviour toward their children due to concern for their children’s safety or health [11]. Parent *overprotection* is sometimes used interchangeably with parent *overcontrol* and *overinvolvement,* but it is a different construct. *Overcontrol* is where parents help or interfere with children’s behaviour without considering children’s desires or interests, and *overinvolvement* is a broader involvement construct consisting of parents’ overprotection and overcontrol [11, 12]. It is hypothesised that parent overprotection may limit a child’s opportunity to face feared situations, learn from their experience, and develop self-efficacy [13]. It has also been hypothesised that parental overprotection may be a response to child anxiety (e.g., [14]) highlighting the importance of reliable measures that can be used to test directional hypotheses.

Several scales have been developed to measure parent overprotection however many existing measures have limitations due to poor internal consistency, a restricted item pool and reliance on child reports. Two of the most commonly used measures are the Egna Minnen Beträffande Uppfostran scale (EMBU) and the Parental Bonding Instrument (PBI). The child-reported EMBU-C consists of 4 factors, emotional warmth, overprotection, favouring subject, and parental rejection [15]. The overprotection factor of the EMBU-C, based on child reports of both mother and father behaviours, has been found to be positively correlated with child trait anxiety [16]. However, the overprotection factor has modest internal consistency (Cronbach’s α = 0.65–0.67 [15],). The brief current form of the Parental Bonding Instrument (PBI-BC [17],) also includes a control/autonomy factor which indicates parents’ tendency to overprotection. Child ratings of both mother and father control/autonomy have been found to significantly correlate with child social anxiety [17]. However the control/autonomy factor only includes two items that assess overprotection (i.e. ‘My mother tries to control everything I do’,’My mother treats me like a baby and tries to protect me from everything’) and these items do not address specific overprotective behaviours. Furthermore it is unclear to what extent anxiety influences children’s perceptions of and reports of parental overprotection which introduces challenges with interpretation of findings.

Although some scales measure parents’ perspectives of overprotective behaviour, these scales have mostly been designed to measure parent overprotection in relation to children/adolescents over 7 years old and not in relation to younger children. For example, a parent-reported version of the EMBU (EMBU-P) was validated with parents of undergraduate students (mean age of 20.33 years; [18]) and a later study implemented the EMBU-P with parents of children from 7 to 18 years old who had been diagnosed with obsessive–compulsive disorder [19]. This study showed that the EMBU-P consists of the same 4 factors as the EMBU-C with good internal consistency, but the study did not examine its validity. Measures of parents’ perception of overprotective behaviour toward young children remain limited despite their potential utility for early detection of risk factors for anxiety disorders.

There are several scales to measure parents’ perspectives of overprotection toward young children, such as Overinvolved/Protective Parenting Scale, New Friends Vignettes, Attitudes about Parenting Strategies for Anxiety, etc. [20]. One scale that has been widely used in clinical studies to measure parents’ views of their behaviour toward their preschool-aged children is the Parent Overprotection Measure (POM [21, 22],). The POM has been found to have high internal consistency, test–retest reliability, and construct validity, and significantly correlates with child anxiety symptoms but not most externalising symptoms [23]. POM scores measured when children were aged 3–5 years were also longitudinally associated with child anxiety symptoms a year later [24]. However, another study conducted with parents of older children (7 to 12 years) only found a significant association between POM and maternal anxiety symptoms and not child anxiety symptoms [21], suggesting that parental overprotection, as measured by the POM, may principally be associated with anxiety in preschool-aged children. Taken together, POM is a promising scale to assess parent overprotection toward preschool-aged children. However, previous studies have only evaluated the POM total score, and the factor structure has not been examined to confirm whether the POM is measuring a unitary construct of overprotection or consists of several underlying dimensions which might function differently from each other.

Previous research has highlighted that some parental behaviours are interpreted differently in different cultures so it is important that measures are evaluated in distinct cultural contexts. Notably, the overprotection factor in the Japanese translation of the EMBU-C loaded on both control and care dimensions, whereas the overprotection factor in the English EMBU-C showed high loading only on control dimensions [25, 26]. Also, Japanese parents may typically be more heavily involved in aspects of their children’s behaviour than Western parents. For example, previous research has indicated that American mothers tend to interpret children’s demanding behaviours as attention-seeking, while Japanese mothers interpret them as a need for security and interdependence [27]. This different interpretation may cause parents to respond in different ways to their children’s behaviour, with Japanese parents potentially more likely to respond to children’s demanding behaviour with protective behaviours. These cultural differences indicate that parenting measures collected in Japan might have different psychometric properties to English-language parenting measures used in Western countries.

The purpose of this study was to translate the English version of the POM into Japanese and examine its factor structure, reliability, and validity based on both mother and father reports. First, we randomly split the total sample of children into two groups and conducted an exploratory factor analysis (EFA) of the Japanese translation of POM (in random group 1), followed by a confirmatory factor analysis (CFA) to confirm the factor structure (in random group 2). Second, to assess psychometric equivalence across mother and father reports, we examined measurement invariance of the mother and father reported Japanese POM. Since traditional gender roles might influence the association between parent overprotection and offspring anxiety [28], we examined the reliability and validity of mother and father reports separately. Third, we calculated the reliability of the mother and father reported Japanese translation of the POM separately by estimating McDonald’s ω coefficients. Fourth, we examined the association between the mother and father reported Japanese translation of the POM and child/parent anxiety symptoms, which is related construct to parent overprotection. We hypothesised that the POM would be weakly correlated with both child and parent anxiety symptoms (*r* ≥ 0.20), referring to the correlation coefficient between the mother/father reported POM and child/parent anxiety symptoms in previous studies [21, 23]. Last, we hypothesised that the correlation between the POM and externalising symptoms (hyperactivity-inattention and conduct problems) would be weak (*r* ≤ 0.10), indicating discriminant validity of the Japanese translation of the POM. The cut-off point was set at 0.10 based on the correlation coefficient between the mother/father reported POM and externalising symptoms in a previous study [23].

## Methods

### Procedure

We collected data on measures online via a research company, Rakuten Insight, in Japan. Rakuten Insight sent an invitation for research participation to members of the general population living in Japan who had registered on their research panel. Participants received points that can be used with Rakuten services. Rakuten Insight controls data quality by removing data with short response time and using dummy questions that require particular answers. Participants were parents of children from the community; there were no specific inclusion requirements in relation to their child’s anxiety. Recruitment continued until 190 mothers and 190 fathers were recruited. We obtained ethical approval from the Ethics Committee of the Chiba University Graduate School of Medicine (study number: M10639). The study protocol of the current study was uploaded on the Open Science Framework before data collection (https://osf.io/c56v7).

### Participants

We collected data from parents of 380 children aged 4 to 7 years (190 mothers and 190 fathers). When participating parents had more than one child in the relevant age range, they were asked to choose one of their children at random and answer accordingly. One parent per child was asked to complete questionnaires. Parents’ and children’s characteristics are shown in Table 1.

**Table 1:** The children and parents’ characteristics

Characteristics		
Total sample (*n* = 380)		
Child		
Age: Mean (SD)		5.49 (1.07)
Gender: n (%)	Male	199 (52.37)
	Female	181 (47.63)
Parent		
Age: Mean (SD)		40.20 (5.84)
Marital status: n (%)	Married	359 (94.47)
	Single	3 (0.79)
	Other	18 (4.74)
Mother reported sample (*n* = 190)		
Child		
Age: Mean (SD)		5.39 (1.05)
Gender: n (%)	Male	91 (47.89)
	Female	99 (52.11)
Mother		
Age: Mean (SD)		38.06 (4.50)
Marital status: n (%)	Married	176 (92.63)
	Single	1 (0.52)
	Other	13 (6.84)
Father reported sample (*n* = 190)		
Child		
Age: Mean (SD)		5.60 (1.08)
Gender: n (%)	Male	108 (56.84)
	Female	82 (43.16)
Father		
Age: Mean (SD)		42.35 (6.23)
Marital status: n (%)	Married	183 (96.31)
	Single	2 (1.05)
	Other	5 (2.63)

### Sample size

The sample size for this study was set at 380 parents based on the minimum required sample size for multiple-group modelling and structural equation modelling (SEM). The rule of thumb for multiple-group modelling is a minimum of 100 observations per group and 10 observations per predictor for SEM [29, 30]. Since the factor structure of POM was not confirmed previously, we followed this rule of thumb to determine the sample size for this study. The POM has 19 items so we aimed to recruit parents of 380 children in total (190 mothers and 190 fathers).

### Measures

#### Preschool Anxiety Scale (PAS)

The PAS is a parent-rated measure comprised of 28 items with a 5-point Likert scale to assess child anxiety symptoms [31]. The PAS has five subscales: generalised anxiety, social anxiety, obsessive–compulsive disorder, physical injury fears, and separation anxiety. The total child anxiety score was based on the sum of all 28 items, with higher scores indicating more anxiety symptoms. The construct validity of the measure has previously been supported by a significant correlation between total score of the PAS and the internalising, but not the externalising scale, of the CBCL [31]. The Japanese translation of the PAS has also shown good construct validity with a significant correlation with CBCL internalising problems [32]. The reliability (McDonald’s *ω* coefficients) of the PAS was 0.93 for the mother report and 0.94 for the father report in this study.

#### Generalised Anxiety Disorder Scale (GAD-7)

The GAD-7 is a self-rated measure to assess adults’ generalised anxiety symptoms. The measure comprises 7 items using a 4-point scale. The GAD-7 has good criterion, construct, factorial, and procedural validity [33]. It also shows good internal consistency (Cronbach’s *α* = 0.92) and test–retest reliability (intraclass correlation = 0.83). A Japanese translation of the GAD-7 has also shown good reliability (Cronbach’s *α* = 0.92) and convergent and discriminant validity [34, 35]. All 7 items of the GAD-7 were added to calculate total parents’ anxiety symptoms. The reliability (McDonald’s *ω* coefficients) of the GAD-7 was 0.88 for both mother and father reports in this study.

#### The Strengths and Difficulties Scale-Parent Version (SDQ-P)

The SDQ-P is a parent-rated measure to assess negative and positive aspects of their child’s behaviour. The measure has 25 items rated from 0 to 2, and it consists of five subscales: emotional symptoms, conduct problems, hyperactivity/inattention, peer relationship problems, and prosocial behaviour [36]. We used the conduct problems and hyperactivity/inattention subscales to test discriminant validity in this study. The Japanese translation of the conduct problems and hyperactivity/inattention subscales have shown good test–retest reliability, convergent validity, and divergent validity [37]. The internal consistency (McDonald’s *ω* coefficients) of the conduct problems was 0.58 for the mother report and 0.62 for the father report in this study. The reliability (McDonald’s *ω* coefficients) of the hyperactivity/inattention was 0.73 for the mother report and 0.68 for the father report in this study.

#### Parent Overprotection Measure (POM)

The POM is a self-rated measure to assess parent overprotection toward their child. The measure consists of 19 items with a 5-point scale from 0 (not at all) to 4 (very much). The POM has good internal consistency (mother: Cronbach’s *α* = 0.87, father: Cronbach’s *α* = 0.86), test–retest reliability (mother:* r* = 0.79, *p* < 0.001, father: *r* = 0.77,* p* < 0.001), and construct validity [23]. The Japanese translation of the POM was developed, referring to the COSMIN checklist manual [38]. First, we obtained permission from the original author of the POM to make the Japanese translation of the POM. Second, two clinical psychologists (SO and HA) translated the original English items separately. Third, an independent translator from an agency back-translated the Japanese translation of the POM into English. Fourth, the original author of the POM (RR) and two researchers with expertise in child anxiety (CC and TR) compared the back-translation and the original items to confirm the appropriateness of the back-translation. Lastly, three clinical psychologists (SO, HA, and TT) and a psychiatrist (ES) discussed the Japanese translation according to feedback from the original author of the POM and the two other child anxiety researchers. Four of the items were amended to reflect the original meaning in the final version of the Japanese-translated POM. The Japanese translation of the POM is available in a supplemental file 1 and Macquarie University website (https://www.mq.edu.au/research/research-centres-groups-and-facilities/centres/lifespan-health-and-wellbeing/our-resources/children-and-teens/_nocache).

### Statistical Analyses

We examined the factor structure, reliability, and discriminant validity of the Japanese translation of POM and its associations with other measures.

Prior to conducting factor analyses, we randomly split mother and father samples separately into two groups using random numbers generated using the RAND function in Microsoft Excel. Then, we combined randomly split groups to form two groups with equal numbers of mothers and fathers. Parents’ and children’s characteristics by the random groups are shown in a supplemental file 2.

We explored the factor structure of POM using exploratory factor analysis (EFA) with Geomin rotation. The EFA was conducted on random group 1. The number of factors was decided based on a scree plot, eigenvalues, factor loading, minimum average partial correlation, and parallel analysis. Then, we confirmed the factor structure of the POM on random group 2 using confirmatory factor analysis (CFA). According to the result of the EFA, we examined one-factor, two-correlated factor, and bi-factor models for the Japanese translation of the POM. We evaluated the model according to the following model fit indices: the comparative fit index (CFI), root mean square error of approximation (RMSEA), and standardized root mean squared residual (SRMR). We followed the following criteria to determine the acceptability of the model fit: CFI > 0.900 is acceptable, CFI > 0.950 is good, RMSEA < 0.800 is acceptable, RMSEA < 0.060 is good, and SRMR < 0.080 is acceptable, SRMR < 0.050 is good [39, 40]. To examine measurement invariance, we conducted multiple group CFA of mother and father reported POM and compared the model fitness index of configural (no constraint), metric (factor loadings constrained), and scalar (factor loadings and intercepts constrained) invariance models. Less than 0.010 change in CFI and 0.015 change in RMSEA was considered as confirmation of the measurement invariance [41]. The maximum likelihood estimation with robust standard errors (MLR) was utilized for EFA, CFA, and multiple group CFA. We chose to use MLR because it is a less biased estimation for ordinal data of a small sample size [42]. The minimum average partial correlation was based on a principal factor method. We estimated McDonald’s Omega and Omega Hierarchical (OmegaH) coefficients of the Japanese translation of the POM to examine its reliability for mother and father reports separately. We then estimated Pearson’s correlation coefficients between the Japanese version of the POM and the PAS, the GAD-7, the hyperactivity-inattention subscale, and the conduct problems subscale of SDQ-P (analysed mother and father reporting separately). To support the discriminant validity of the POM, we expected the correlation coefficient of the POM and the hyperactivity-inattention/conduct problems subscales to show a similar correlation coefficient to the original POM, which was 0.10 or less. The cut-off points for the validities were set based on the correlation coefficient between the mother/father reported POM and each measure in a previous study [21, 23]. EFA, CFA, and multiple group CFA were conducted using Mplus [43], McDonald’s omega and OmegaH were estimated using the Bifactor Indices Calculator [44], and Pearson’s correlation coefficients were estimated using STATA (Stata Corp, College Station).

## Results

### Factor Structure of the POM

EFA was conducted on random group 1 to examine the factor structure of the Japanese translation of the POM. The eigenvalues of the first five factors in the POM were 6.64, 2.31, 1.27, 1.00, and 0.87, which suggests four factors according to the Kaiser criterion. On the contrary, the parallel analysis and minimum average partial correlation suggested two-factor structures for the POM. Since two of the three criteria suggested two factors and factor loadings of three or more factors were not interpretable, we accepted two factors for the POM from the EFA. The factor loadings of each item for two factors are shown in Table 2. Some items showed cross-loadings across the two factors; however, we chose to retain them for the CFA since this may indicate the possibility of a latent single factor.

**Table 2:** The result of EFA for the random split sample 1

Item	Factor loadings	
	F1	F2
Factor 1		
4. I give my child extra attention when he/she clings to me	0.78	− 0.11
2. When playing in a park, I keep my child within a close distance of me (i.e., within about 30m)	0.68	0.00
3. I protect my child from criticism	0.64	0.10
7. I keep a close watch on my child at all times	0.63	0.04
19. I protect my child from his/her fears	0.46	0.43
9. I try to anticipate and avoid situations where my child might do something risky	0.43	0.35
6. I almost always take my child to the doctor if he/she is unwell	0.40	0.05
1. I comfort my child immediately when he/she cries	0.39	0.22
15. I will only leave my child with close friends or relatives if I have to go out	0.30	0.27
Factor 2		
10. I try to protect my child from making mistakes	0.00	0.73
17. I shield my child from conflict	0.14	0.72
14. I am reluctant for my child to play some sports for fear he/she might get hurt	− 0.58	0.71
12. I shelter my child from life’s difficulties	0.13	0.67
13. When away from home I tend to panic if my child is out of my sight, even for a moment	− 0.18	0.64
11. I do not allow my child to climb trees	− 0.24	0.61
18. I do everything possible to protect my child from potential injury	0.26	0.61
8. I tend to be over-protective of my child	0.15	0.40
5. I would not allow my child to go out with family friends if I were not present	0.10	0.38
16. I accompany my child on all outings	0.22	0.36

**Fig. 1:**
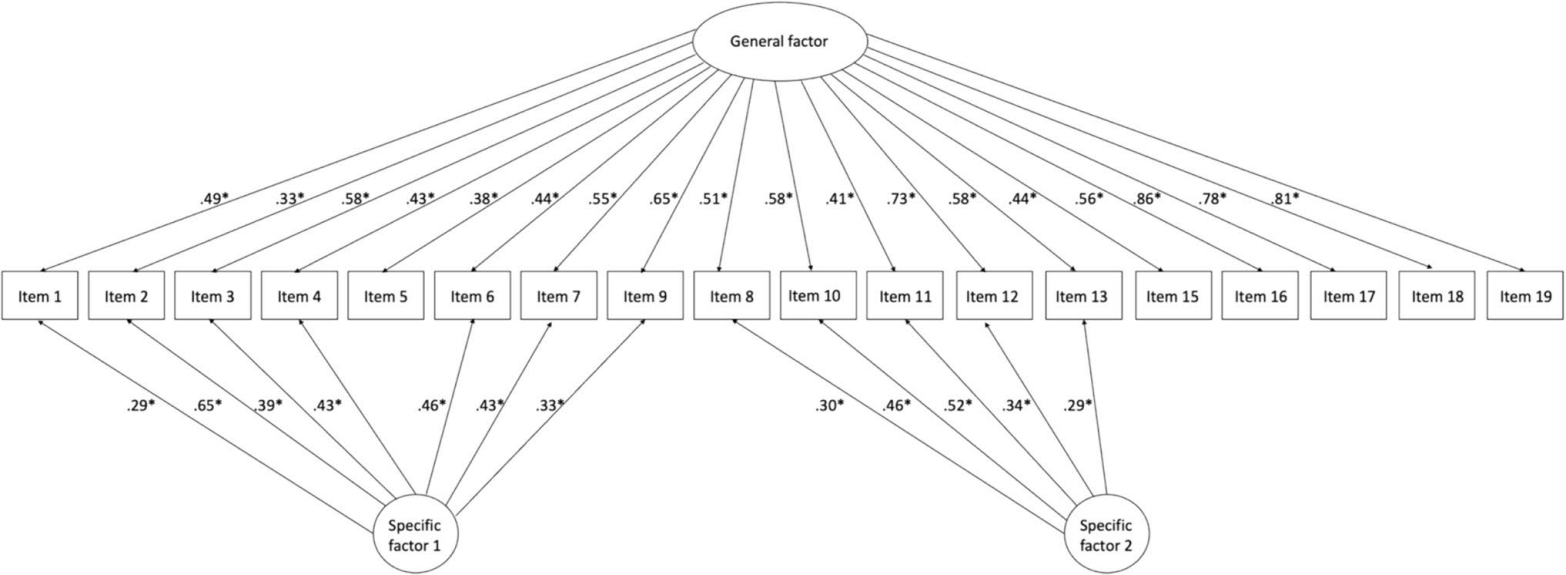
The accepted bi-factor model of the POM for the random split sample 2

To confirm the factor structure of the Japanese translation of the POM, we conducted a CFA on random group 2. We examined the two-factor correlated model with and without cross-loadings according to the results of the EFA. In addition, we examined one-factor and bi-factor models and compared these models to determine the best-fit factor model for the Japanese translation of the POM. We considered the one-factor model and bi-factor model as the comparison model because the original POM study assumed the POM represents general parent overprotection, and our EFA result showed cross-loadings on two factors in some items. The model fit indices for each model are illustrated in Table 3. We determined the best model for the POM based on the fulfilment of cut-off criteria on fit indices and clinical interpretability of the factor structure. Only the bi-factor models showed an acceptable range of fit indices [39, 40]. Item 14 had no significant loading to the general factor in the bi-factor model (Factor loading = 0.16, *p* = 0.115). Therefore, we excluded it from the bi-factor model and reran the bi-factor model. Although all the items loaded significantly to the general factor, items 15 and 19 failed to show significant factor loadings on specific factor 1 (Item 15: factor loading = 0.058, *p* = 0.585, Item 19: factor loading = 0.085, *p* = 0.329) and items 5, 16, 17, and 18 on specific factor 2 (Item 5: factor loading = 0.069, *p* = 0.627, Item 16: factor loading = − 0.026, *p* = 0.835, Item 17: factor loading = 0.164, *p* = 0.120, Item 18: factor loading = 0.170, *p* = 0.127). We also ran the bi-factor model without factor loadings of items 5 and 15 to 19 on the specific factors. We accepted a more parsimonious model for the final model, which is the bi-factor model without the factor loadings of items 5 and 15 to 19 on the specific factors (Fig. 1).

**Table 3:** The model fit indices of the factor structure of the POM for the random split sample 2

Model	χ^2^	df	CFI	RMSEA	SRMR
One-factor model	456.974	152	.772	.103	.093
Two-factor correlated model	373.599	151	.834	.088	.087
Two-factor correlated model with cross-loadings^a^	312.780	148	.877	.077	.078
Bi-factor model	216.316	133	.938	.057	.055
Bi-factor model without item 14	180.834	117	.947	.054	.051
Bi-factor model with restriction^b^	187.696	123	.947	.053	.053

^a^According to the result of EFA (Factor loading .32 or more on both factors), we added the cross-loading of Items 9, 14, and 19 in the model

^b^The bi-factor model without factor loadings of items 5 and 15 to 19 on the specific factors

We conducted multiple group CFA to examine the measurement invariance of the Japanese translation of the POM between mother and father reports. The model fit change between the configural (CFI: 0.909, RMSEA: 0.066) and metric (CFI: 0.903, RMSEA: 0.064) models was 0.006 for CFI and 0.002 for RMSEA. The model fit change between the metric and scalar (CFI: 0.903, RMSEA: 0.064) models was 0.000 for CFI and 0.000 for RMSEA.

### Reliability of the POM

McDonald’s *ω* coefficients were estimated for each factor. Since the final model yielded a bi-factor structure, we also estimated the OmegaH coefficients. The *ω* coefficients were 0.92 for the general factor, 0.84 for the specific factor 1, and 0.79 for the specific factor 2 for the father-reported POM. For the mother-reported POM, the *ω* coefficients were 0.91 for the general factor, 0.82 for the specific factor 1, and 0.79 for the specific factor 2. The OmegaH coefficients for the father-reported POM were 0.83 for the general factor, explaining 75.6% of the total variance, 0.30 for the specific factor 1, explaining 14.8% of the unique variance, and 0.25 for the specific factor 2, explaining 9.6% of the unique variance in the data. The OmegaH coefficients for the mother-reported POM were 0.79 for the general factor, explaining 72.2% of the total variance, 0.43 for the specific factor 1, explaining 19.4% of the unique variance, and 0.24 for the specific factor 2, explaining 8.4% of the unique variance in the data.

### Associations with Other Measures and Discriminant Validity of the POM

The means and standard deviations of each measure for mothers and fathers are shown in Table 4. We estimated Pearson’s correlation coefficients between the general factor and each specific factor of the POM, against PAS, GAD-7, the conduct problems and hyperactivity/inattention subscales of the SDQ-P. The estimated correlation coefficients are shown in Table 5. For the general factor of the POM, no measure exceeded the correlation coefficient of 0.20 in both mother and father reports. For the specific factors, the correlation coefficients exceeded 0.20 for the specific factor 2 and PAS in both the mother and father reports, and the specific factor 2 and GAD-7 in the father report. In addition, a significant and greater than 0.10 correlation was obtained between the conduct problems and specific factor 1 in both the mother and father reports, and the conduct problems and specific factor 2 in the father report.

**Table 4:** Mean and SD of each measure for mother and father reports

	Mother (*n* = 190)		Father (*n* = 190)	
	Mean	SD	Mean	SD
POM				
General factor	39.53	11.22	36.28	11.21
Specific factor 1	17.78	4.73	16.43	4.84
Specific factor 2	8.62	3.59	8.11	3.55
PAS	29.92	16.17	32.95	16.71
GAD-7	4.00	4.36	3.33	3.92
SDQ-P				
Conduct problems	2.48	1.71	2.43	1.68
Hyperactivity/inattention	4.01	2.27	4.20	2.07

*POM* parent overprotection measure, *PAS* preschool anxiety scale, *GAD-7* generalised anxiety disorder scale, *SDQ-P* the strength and difficulties scale-parent version

**Table 5:** The correlation coefficients between the POM and other measures for mother and father reports

	Mother			Father		
	POM			POM		
	General factor	Specific factor 1	Specific factor 2	General factor	Specific factor 1	Specific factor 2
PAS	.15*	− .01	.27***	.05	− .17*	.32***
GAD-7	.12	.01	.15*	.14*	− .04	.28**
Conduct problems	− .06	− .18**	.06	− .03	− .18*	.15*
Hyperactivity/Inattention	.00	− .11	.09	.02	− .03	.07

*POM* parent overprotection measure, *PAS* preschool anxiety scale, *GAD-7* generalised anxiety disorder scale

***p < .001, **p < .01, *p < .05

## Discussion

The purpose of the study was to evaluate the factor structure, reliability, and validity of the mother and father reports of a Japanese translation of the Parent Overprotection Measure (POM). We examined the factor structure of the POM using both exploratory and confirmatory perspectives. The Japanese translation of the POM yielded a bi-factor structure, with the general factor explaining most variance in the data. The bi-factor model exceeded the acceptable level of CFI, RMSEA, and SRMR [39, 40]. Measurement invariance between mother and father reports was supported.

Previous studies using the POM have utilised a total score in accordance with the assumption of unidimensionality (e.g., [23]). This is the first study to empirically assess the factor structure of the POM, and the result was partially consistent with this assumption. However, not all the variance in the POM was explained by the general factor, and residual variances were explained by two specific factors, which we consider to reflect care/attention for specific factor 1 and control/prevention for specific factor 2. The nature of the specific factors was determined based on the characteristics of the items in each factor and the directions of correlations with measures of anxiety and externalising symptoms. Two possible explanations for structure of the POM found here are that (i) parent overprotection is a parental behaviour that overlaps with the parent’s control and care behaviours. Previous studies often use overprotection and overcontrol interchangeably, and overprotection is highly associated with parental warmth, which may indicate some overprotective behaviours also function as control and care behaviours [15, 45],and (ii) the Japanese translation of the POM may indicate a culture-specific structure. A previous Japanese study that found the overprotection factor in the EMBU-C loaded on higher factors of control and care dimensions [26], whereas the EMBU-C only loaded on the control dimension among a Dutch sample [25] highlighting the potential for cross-cultural differences. A future study is warranted to examine the factorial invariance or variance of the Japanese and English versions of the POM in different cultures. Having said this, although the general factor explained most of the variance in the POM, and the care/attention and control/prevention factors only explained 14.8–19.4% and 8.4–9.6% of the variance, respectively, the McDonald’s omega of the general overprotection, care/attention, and control/prevention factors of the POM were over 0.70, indicating sufficient reliability [46]. Since general overprotection, care/attention, and control/prevention have sufficient reliability and relate differently to other measures, it may be beneficial to consider each factor separately to understand the parent’s characteristics from a broad perspective.

Our hypothesis that the POM would be correlated with both child and parent anxiety symptoms was not supported. The correlation between the general factor of the POM and child or parent anxiety symptoms did not exceed 0.20 for either mother or father reports. This result is inconsistent with previous studies which found correlations of greater than 0.20 between the POM and child and parent anxiety symptoms [21, 23]. However, the correlation between the control/prevention factor and child anxiety was over 0.20 for both mother and father reports and the correlation between control/prevention factors and parent anxiety symptoms was over 0.20 for father reports. There are two possible explanations for the weak correlation for the general factor and moderate correlation for the control/prevention factor. One is that, given the best-fit model of the POM was the bi-factor model, all the variance in the POM might not be explained by one general factor. As found by Chevrier et al. [47], different sorts of parent overprotection behaviours may be differentially associated with anxiety symptoms. Another possible reason is that the cultural norms and interpretation of parent overprotection may differ between cultures. Enmeshment between parent and child is valued and often interpreted as an expression of care in Asian cultures [28, 48]. This may indicate that some of the general parent overprotection behaviours were interpreted as care and, as such, had less association with child and parent anxiety symptoms. Further studies are warranted to explore these possible reasons for its weak correlation with child and parent anxiety symptoms.

Our hypothesis that the correlation between the POM and externalising symptoms (hyperactivity-inattention and conduct problems) would be weak (*r* ≤ 0.10) was supported. The correlation between the general factor and conduct problems and hyperactivity/inattention was less than 0.10, consistent with the previous findings [23]. In contrast, conduct problems showed a significant and greater than 0.10 negative correlation with the care/attention factor for both the mother and father reports, and a significant and greater than 0.10 positive correlation with the control/prevention factor for father reports. This may suggest that the two specific factors may act slightly differently from overall parent overprotection. In line with our suggestion that among our Japanese population the subscales may reflect care/attention and control, a previous meta-analysis found that parental warmth is negatively associated with a child’s externalising problems, while parental psychological and harsh control is positively associated with a child’s externalising problems [49].

### Limitations

There were several limitations in this study. First, the assessment of validity relied completely on parent-reported measures of externalising symptoms. This study did not include another measure of parent overprotection, such as observational measures. In addition, the internal consistency of the subscales of the SDQ-P was low in this study. Second, test–retest reliability was not examined in the current study and this will be important to address in future studies. Third, this study purposefully included a Japanese sample however whether the factor structure can be generalised to other cultures is unclear. Given the current findings suggesting potential cross-cultural variation, it will be important to examine the factor structure and measurement invariance of the POM in other cultures in future studies. Finally, this study collected data from only one parent per child. It may be beneficial for future studies to collect both mother and father data for each child to compare parents’ overprotection of the same child, to help elucidate child versus parent effects.

## Summary

The development of the Japanese translation of the POM contributes to the study of parent overprotection in East Asia. The Japanese translation of the POM may be able to measure parents’ perspectives of their overprotection towards children in Japan and the development of the Japanese translation is an important first step toward the progress of understanding the function of parent overprotection in East Asian cultures. This study found that the association between the Japanese translation of the POM and child anxiety symptoms is very weak. Whether this very weak association is characteristic of the general overprotection factor of the POM or is due to cultural differences in the nature of the overprotection is unclear. Future research is needed to clarify the characteristics of the Japanese translation of the POM and the cultural difference of the overprotection.

## Supplementary Information

Below is the link to the electronic supplementary material.

Supplementary file1 (DOCX 315 KB)

Supplementary file2 (DOCX 15 KB)

## Data Availability

The datasets generated and/or analysed during the current study are available from the corresponding author upon reasonable request.

## References

[1] Essau CA, Conradt J, Petermann F (2000) Frequency, comorbidity, and psychosocial impairment of anxiety disorders in German adolescents. J Anxiety Disord 14:263–279. 10.1016/s0887-6185(99)00039-0 10868984 10.1016/s0887-6185(99)00039-0

[2] Solmi M, Radua J, Olivola M, Croce E, Soardo L, de Pablo GS et al (2022) Age at onset of mental disorders worldwide: large-scale meta-analysis of 192 epidemiological studies. Mol Psych 27:281–295. 10.1038/s41380-021-01161-7 10.1038/s41380-021-01161-7 PMC8960395 34079068

[3] Pollard J, Reardon T, Williams C, Creswell C, Ford T, Gray A et al (2023) The multifaceted consequences and economic costs of child anxiety problems: a systematic review and meta-analysis. JCPP Adva 3(3):e12149. 10.1002/jcv2.12149 10.1002/jcv2.12149 PMC10501703 37720587

[4] Gazelle H, Rubin KH (2010) Social anxiety in childhood: bridging developmental and clinical perspectives. New Dir Child Adolesc Dev 2010(127):1–16. 10.1002/cd.259 20205182 10.1002/cd.259 PMC3733263

[5] Ishikawa S (2015) A cognitive-behavioral model of anxiety disorders in children and adolescents. Jpn Psychol Res 57(3):180–193. 10.1111/jpr.12078

[6] Rapee RM, Creswell C, Kendall PC, Pine DS, Waters AM (2023) Anxiety disorders in children and adolescents: an overview of the literature. Behav Res Ther 168:104376. 10.1016/j.brat.2023.104376 37499294 10.1016/j.brat.2023.104376

[7] Murray L, Creswell C, Cooper PJ (2009) The development of anxiety disorders in childhood: an integrative review. Psychol Med 39(9):1413–1423. 10.1017/S0033291709005157 19215631 10.1017/S0033291709005157

[8] Yap MBH, Jorm AF (2015) Parental factors associated with childhood anxiety, depression, and internalizing problems: a systematic review and meta-analysis. J Affect Disord 175:424–440. 10.1016/j.jad.2015.01.050 25679197 10.1016/j.jad.2015.01.050

[9] McLeod BD, Wood JJ, Weisz JR (2007) Examining the association between parenting and childhood anxiety: a meta-analysis. Clin Psychol Rev 27:155–172. 10.1016/j.cpr.2006.09.002 17112647 10.1016/j.cpr.2006.09.002

[10] Rapee RM (1997) Potential role of childrearing practices in the development of anxiety and depression. Clin Psychol Rev 17:47–67. 10.1016/S0272-7358(96)00040-2 9125367 10.1016/s0272-7358(96)00040-2

[11] Möller EL, Nikolić M, Majdandžić M, Bögels SM (2016) Associations between maternal and paternal parenting behaviors, anxiety and its precursors in early childhood: a meta-analysis. Clin Psychol Rev 45:17–33. 10.1016/j.cpr.2016.03.002 26978324 10.1016/j.cpr.2016.03.002

[12] Majdandžić M, Möller EL, de Vente W, Bögels SM, van den Boom DC (2014) Fathers’ challenging parenting behavior prevents social anxiety development in their 4-year-old children: a longitudinal observational study. J Abnorm Child Psychol 42(2):301–310. 10.1007/s10802-013-9774-4 23812638 10.1007/s10802-013-9774-4

[13] Rapee RM (2001) The development of generalized anxiety. In: Vasey MW, Dadds MR (eds) The developmental psychopathology of anxiety. Oxford University Press, New York, pp 481–503

[14] Hudson JL, Doyle AM, Gar N (2009) Child and maternal influence on parenting behavior in clinically anxious children. J Clin Child Adolesc Psychol 38(2):256–262. 10.1080/15374410802698438 19283603 10.1080/15374410802698438

[15] Castro J, Toro J, Van der Ende J, Arrindell WA (1993) Exploring the feasibility of assessing perceived parental rearing styles in Spanish children with the EMBU. Int J Soc Psychiatry 39(1):47–57. 10.1177/002076409303900105 8478163 10.1177/002076409303900105

[16] Markus MT, Lindhout IE, Boer F, Hoogendijk THG, Arrindell WA (2003) Factors of perceived parental rearing styles: the EMBU-C examined in a sample of Dutch primary school children. Personality Individ Differ 34(3):503–519. 10.1016/S0191-8869(02)00090-9

[17] Klimidis S, Minas IH, Ata AW (1992) The PBI-BC: a brief current form of the parental bonding instrument for adolescent research. Compr Psychiatry 33(6):374–377. 10.1016/0010-440x(92)90058-x 1451449 10.1016/0010-440x(92)90058-x

[18] Castro J, de Pablo J, Gómez J, Arrindell WA, Toro J (1997) Assessing rearing behaviour from the perspective of the parents: a new form of the EMBU. Soc Psychiatry Psychiatr Epidemiol 32:230–235. 10.1007/BF00788243 9184469 10.1007/BF00788243

[19] Mathieu SL, Conlon EG, Waters AM, Farrell LJ (2020) Perceived parental rearing in pediatric obsessive-compulsive disorder: examining the factor structure of the EMBU child and parent versions and associations with OCD symptoms. Child Psychiatry Hum Dev 51:956–968. 10.1007/s10578-020-00979-6 32146572 10.1007/s10578-020-00979-6

[20] Lohman A, Bayer JK (2020) Overinvolved/protective parenting questionnaires for children: a systematic review in the field of internalizing problems. Int J Mental Health Promotion 22(4):203–219. 10.32604/IJMHP.2020.011789

[21] Clarke K, Cooper P, Creswell C (2013) The parental overprotection scale: associations with child and parental anxiety. J Affect Disord 151(2):618–624. 10.1016/j.jad.2013.07.007 23916305 10.1016/j.jad.2013.07.007 PMC3808745

[22] Reardon T, Dodd H, Hill C, Jasper B, Lawrence PJ, Morgan F (2022) Minimising Young Children’s Anxiety Through Schools (MY-CATS): protocol for a cluster randomised controlled trial to evaluate the effectiveness and cost-effectiveness of an online parent-led intervention compared with usual school practice for young children identified as at risk for anxiety disorders. Trials 23:149. 10.1186/s13063-022-06010-8 35168635 10.1186/s13063-022-06010-8 PMC8848959

[23] Edwards SL, Rapee RM, Kennedy S (2008) Psychometric properties of a parent-report measure of overprotection in preschool-aged children. Unpublished manuscript.

[24] Edwards SL, Rapee RM, Kennedy S (2010) Prediction of anxiety symptoms in preschool-aged children: examination of maternal and paternal perspectives. J Child Psychol Psychiatry 51:313–321. 10.1111/j.1469-7610.2009.02160.x 19769584 10.1111/j.1469-7610.2009.02160.x

[25] Muris P, Meesters C, van Brakel A (2003) Assessment of anxious rearing behaviors with a modified version of “Egna Minnen Beträffande Uppfostran” questionnaire for children. J Psychopathol Behav Assess 25:229–237. 10.1023/A:1025894928131

[26] Nishikawa S, Sundbom E, Hägglöf B (2010) Influence of perceived parental rearing on adolescent self-concept and internalizing and externalizing problems in Japan. J Child Fam Stud 19(1):57–66. 10.1007/s10826-009-9281-y

[27] Rothbaum F, Kakinuma M, Nagaoka R, Azuma H (2007) Attachment and amae: parent-child closeness in the United States and Japan. J Cross Cult Psychol 38(4):465–486. 10.1177/0022022107302315

[28] de Roo M, Veenstra R, Kretschmer T (2022) Internalizing and externalizing correlates of parental overprotection as measured by the EMBU: a systematic review and meta-analysis. Soc Dev 31(4):962–983. 10.1111/sode.12590 36588978 10.1111/sode.12590 PMC9790597

[29] Kline RB (2005) Principles and practice of structural equation modeling, 2nd edn. Guilford, New York

[30] Nunnally JC (1967) Psychometric theory. McGraw-Hill, New York

[31] Spence SH, Rapee R, McDonald C, Ingram M (2001) The structure of anxiety symptoms among preschoolers. Behav Res Ther 39(11):1293–1316. 10.1016/s0005-7967(00)00098-x 11686265 10.1016/s0005-7967(00)00098-x

[32] Nakagawa Y, Fujiu H (2015) Development of Japanese version of preschool anxiety scale. The Journal of Child Health 74(2):247–253

[33] Spitzer RL, Kroenke K, Williams JB, Löwe B (2006) A brief measure for assessing generalized anxiety disorder: the GAD-7. Arch Intern Med 166(10):1092–1097. 10.1001/archinte.166.10.1092 16717171 10.1001/archinte.166.10.1092

[34] Doi S, Ito M, Takebayashi Y, Muramatsu K, Horikoshi M (2018) Factorial validity and invariance of the 7-item Generalized Anxiety Disorder Scale (GAD-7) among populations with and without self-reported psychiatric diagnostic status. Front Psychol 9:1741. 10.3389/fpsyg.2018.01741 30283386 10.3389/fpsyg.2018.01741 PMC6157449

[35] Muramatsu K, Muramatsu Y, Miyaoka H, Fuse K, Yoshimine F, Hosaka M, Kutsumi R (2009) Validation and utility of a Japanese version of the GAD-7. In: 20th World Congress on psychosomatic medicine. Italy.

[36] Goodman R (2001) Psychometric properties of the strengths and difficulties questionnaire. J Am Acad Child Adolesc Psychiatry 40(11):1337–1345. 10.1097/00004583-200111000-00015 11699809 10.1097/00004583-200111000-00015

[37] Moriwaki A, Kamio Y (2014) Normative data and psychometric properties of the strengths and difficulties questionnaire among Japanese school-aged children. Child Adolesc Psychiatry Ment Health 8(1):1. 10.1186/1753-2000-8-1 24444351 10.1186/1753-2000-8-1 PMC3903008

[38] Mokkink LB, Terwee CB, Patrick DL, Alonso J, Stratford PW, Knol DL, Bouter LM, de Vet HC (2012) COSMIN checklist manual. University Medical Center, Amsterdam

[39] Hu L, Bentler PM (1999) Cutof criteria for ft indexes in covariance structure analysis: conventional criteria versus new alternatives. Struct Equ Modeling 6(1):1–55. 10.1080/10705519909540118

[40] Schumacker RE, Lomax RG (2010) A beginner’s guide to structural equation modeling, 3rd edn. Routledge/Taylor & Francis Group

[41] Chen FF (2007) Sensitivity of goodness of fit indexes to lack of measurement invariance. Struct Equ Modeling 14(3):464–504. 10.1080/10705510701301834

[42] Li CH (2016) Confirmatory factor analysis with ordinal data: comparing robust maximum likelihood and diagonally weighted least squares. Behav Res Methods 48(3):936–949. 10.3758/s13428-015-0619-7 26174714 10.3758/s13428-015-0619-7

[43] Muthén LK, Muthén BO (1998–2017) Mplus user’s guide, 8th edn. Muthén & Muthén

[44] Dueber DM (2017) Bifactor indices calculator: a Microsoft Excel-based tool to calculate various indices relevant to bifactor CFA models. 10.13023/edp.tool.01

[45] Taylor CT, Alden LE (2006) Parent overprotection and interpersonal behavior in generalized social phobia. Behav Ther 37(1):14–24. 10.1016/j.beth.2005.03.001 16942957 10.1016/j.beth.2005.03.001

[46] McNeish D (2018) Thanks coefficient alpha, we’ll take it from here. Psychol Methods 23(3):412–433. 10.1037/met0000144 28557467 10.1037/met0000144

[47] Chevrier B, Soenens B, Zimmermann G, Skhirtladze N, Petegem SV (2023) The psychometric qualities of a short version of the multidimensional overprotective parenting scale. Eur J Dev Psychol 20(3):550–566. 10.1080/17405629.2022.2079630

[48] Rothbaum F, Rosen K, Ujiie T, Uchida N (2002) Family systems theory, attachment theory, and culture. Fam Process 41(3):328–350. 10.1111/j.1545-5300.2002.41305.x 12395563 10.1111/j.1545-5300.2002.41305.x

[49] Pinquart M (2017) Associations of parenting dimensions and styles with externalizing problems of children and adolescents: an updated meta-analysis. Dev Psychol 53(5):873–932. 10.1037/dev0000295 28459276 10.1037/dev0000295

