# Supplementary material for: Psychometric Properties of the Japanese Translation of the Parent Overprotection Measure for Mother and Father Reports
Source: Child Psychiatry Hum Dev. 2024 Sep 9;57(3):905–15. doi: 10.1007/s10578-024-01753-8 (PMC13201325; doi:10.1007/s10578-024-01753-8)
Supplement: Supplementary file 1 — Supplementary file1 (DOCX 315 KB) [file 10578_2024_1753_MOESM1_ESM.docx]

Supplemental file 1 The Japanese translation of the Parent Overprotection Scale

それぞれの質問について、「0. 全く」から「4. とても」の間で、最も当てはまるものに○をつけてください。
