# Supplementary material for: Psychometric Properties of the Japanese Translation of the Parent Overprotection Measure for Mother and Father Reports
Source: Child Psychiatry Hum Dev. 2024 Sep 9;57(3):905–15. doi: 10.1007/s10578-024-01753-8 (PMC13201325; doi:10.1007/s10578-024-01753-8)
Supplement: Supplementary file 2 — Supplementary file2 (DOCX 15 KB) [file 10578_2024_1753_MOESM2_ESM.docx]

Supplemental file 2 The children and parents’ characteristics by random split groups

| Characteristics |  |  |
| --- | --- | --- |
| Random group 1 (n = 190) |  |  |
| Child |  |  |
| Age: Mean (SD) |  | 5.51 (1.97) |
| Gender: n (%) | Male | 92 (48.42) |
|  | Female | 98 (51.58) |
| Parent |  |  |
| Age: Mean (SD) |  | 40.16 (6.32) |
| Gender: n (%) | Male | 95 (50) |
|  | Female | 95 (50) |
| Marital status: n (%) | Married | 180 (94.74) |
|  | Single | 0 (0) |
|  | Other | 10 (5.26) |
| Random group 2 (n = 190) |  |  |
| Child |  |  |
| Age: Mean (SD) |  | 5.47 (1.07) |
| Gender: n (%) | Male | 107 (56.32) |
|  | Female | 83 (43.68) |
| Parent |  |  |
| Age: Mean (SD) |  | 40.24 (5.32) |
| Gender: n (%) | Male | 95 (50) |
|  | Female | 95 (50) |
| Marital status: n (%) | Married | 179 (94.21) |
|  | Single | 3 (1.58) |
|  | Other | 8 (4.21) |
